# Study protocol: Cost effectiveness of two strategies to implement the NVOG guidelines on hypertension in pregnancy: An innovative strategy including a computerised decision support system compared to a common strategy of professional audit and feedback, a randomized controlled trial

**DOI:** 10.1186/1748-5908-5-68

**Published:** 2010-09-06

**Authors:** Susanne HE Luitjes, Maurice GAJ Wouters, Arie Franx, Hubertina CJ Scheepers, Veerle MH Coupé, Huub Wollersheim, Eric AP Steegers, Martijn P Heringa, Rosella PMG Hermens, Maurits W van Tulder

**Affiliations:** 1 Department of Obstetrics and Gynaecology, VU University Medical Centre, PO Box 7057, 1007 MB Amsterdam, Netherlands; 2 Department of Health Sciences, Faculty of Earth & Life Sciences, EMGO+ Institute for Health and Care Research, VU University, De Boelelaan 1085, 1081 HV Amsterdam, Netherlands; 3 Department of Obstetrics and Gynaecology, St. Elisabeth Hospital, PO Box 90515, 5000 LC Tilburg, Netherlands; 4 Department of Obstetrics and Gynaecology, University Hospital Maastricht, PO Box 5800, 6202 AZ Maastricht, Netherlands; 5 Department of Epidemiology and Biostatistics, VU University Medical Centre, PO Box 7057, 1007 MB Amsterdam, Netherlands; 6 Scientific Institute for Quality of Health Care (IQ healthcare) Radboud University Nijmegen Medical Centre, PO Box 9101, 6500 HB Nijmgen, Netherlands; 7 Department of Obstetrics and Gynaecology, Erasmus Medical Centre, PO Box 2040, 3000 CA Rotterdam, Netherlands; 8 Department of Obstetrics and Gynaecology, University Medical Centre Utrecht, PO Box 85500, 3508 GA Utrecht, Netherlands

## Abstract

**Background:**

Hypertensive disease in pregnancy remains the leading cause of maternal mortality in the Netherlands. Seventeen percent of the clinical pregnancies are complicated by hypertension and 2% by preeclampsia. The Dutch Society of Obstetrics and Gynaecology (NVOG) has developed evidence-based guidelines on the management of hypertension in pregnancy and chronic hypertension. Previous studies showed a low adherence rate to other NVOG guidelines and a large variation in usual care in the different hospitals. An explanation is that the NVOG has no general strategy of practical implementation and evaluation of its guidelines. The development of an effective and cost effective implementation strategy to improve adherence to the guidelines on hypertension in pregnancy is needed.

**Methods/Design:**

The objective of this study is to assess the cost effectiveness of an innovative implementation strategy of the NVOG guidelines on hypertension including a computerised decision support system (BOS) compared to a common strategy of professional audit and feedback. A cluster randomised controlled trial with an economic evaluation alongside will be performed. Both pregnant women who develop severe hypertension or pre-eclampsia and professionals involved in the care for these women will participate. The main outcome measures are a combined rate of major maternal complications and process indicators extracted from the guidelines. A total of 472 patients will be included in both groups. For analysis, descriptive as well as regression techniques will be used. A cost effectiveness and cost utility analysis will be performed according to the intention-to-treat principle and from a societal perspective. Cost effectiveness ratios will be calculated using bootstrapping techniques.

## Background

Hypertension is a common complication of pregnancy. Seventeen percent of the clinical pregnancies are complicated by hypertension and 2% by preeclampsia [1-6]. Severe hypertension and preeclampsia (hypertension and proteinuria) pose an increased risk for morbidity and mortality to both the mother and the foetus. Major maternal complications (*e.g*., eclampsia, HELLP syndrome, pulmonary oedema, placental abruption, liver haematoma, severe infectious or thrombotic morbidity, encephalopathy) were recently reported in 74 of 216 (34%) Dutch pregnant women with severe hypertension or preeclampsia [7,8]. The Dutch trends in maternal morbidity due to severe hypertension and preeclampsia are currently a reason for great concern [9]. Hypertensive disease in pregnancy remains the leading cause of maternal mortality in the Netherlands, followed by thromboembolism and obstetric haemorrhage. Fifty-one percent of the total number of maternal deaths is a result of hypertensive complications in pregnancy. The maternal mortality ratio (MMR; deaths per 100,000 live births) from hypertensive disease in pregnancy increased from 2.7 in 1983 to 1992 to 4.0 in 1993 to 2002 [10], a major difference with surrounding countries. In the UK, maternal mortality due to hypertensive disease ranks fourth place-- with a MMR of 0.7 -- following thromboembolism, obstetric haemorrhage, and death during first trimester (ectopic pregnancies, miscarriage, and termination of pregnancy) [11].

It is thought that a high degree of these maternal complications results from suboptimal or insufficient treatment [12,13]. A recent study of the Dutch Committee on Maternal Death showed that in 96% of the cases of maternal death due to hypertensive disease several factors of substandard care were present. For instance, in 85% of this patient group, treatment of severe hypertension was inadequate. In many cases, the decision to start therapy was either made too late or not at all [14]. Therefore, management of hypertension is an important part of care for pregnant women for which improvement is necessary. To support their members, the Dutch Society of Obstetrics and Gynaecology (NVOG) has developed evidence-based clinical guidelines on the management of hypertension in pregnancy [15] and chronic hypertension in pregnancy [16]. However, these guidelines are not yet implemented. The NVOG has no general strategy of practical implementation and evaluation of its guidelines, and has only just begun with a policy of hospital visitations in order to improve quality of clinical care. In order to reduce maternal complications, an implementation strategy to improve adherence to the guidelines on hypertension in pregnancy is necessary

Original studies and systematic reviews about the effectiveness of different interventions to change clinical practice show that a widely used strategy for implementing guidelines consists of audit and feedback about actual performance. This strategy has shown to be effective, but feedback is often recommended in combination with other strategies [17]. In a recent systematic review, a major benefit of implementing health information technology on increased adherence to guideline-based care was demonstrated, including hypertension [18]. Different health information technology systems were reviewed. Most of them included a decision support system, and some of them integrated clinical guidelines [19]. In case of hypertension in pregnancy, an implementation strategy including a computerised decision support system (BOS) may lead to a higher compliance to the guidelines' recommendations and thus a lower rate of maternal complications and their costs. This will be tested in this study.

It is expected that an increased adherence to the guidelines' recommendations will reduce the number of major maternal complications. By reducing the number of major maternal complications, we expect a reduction in health care costs (*e.g*., costs of hospitalisation, medication), costs in other sectors, patient and family costs, and costs of production losses [20]. Estimating the size of this cost reduction is an essential part of this study.

## Objectives

The objective of this study is to assess the effectiveness and cost effectiveness of an innovative implementation strategy of the NVOG guidelines on hypertension, including a computerised decision support system compared to a common strategy of professional audit and feedback.

The research questions are:

1. To what extent is an innovative strategy using a computerised decision support system cost effective compared to a common strategy of professional audit and feedback in implementing the NVOG guidelines on hypertension in pregnancy?

2. What is the feasibility of the two implementation strategies?

3. What is the cost effectiveness of the two strategies to implement the guidelines on hypertension in pregnancy in clinical practice?

## Methods/Design

### Study design

A cluster randomised controlled trial with an economic evaluation alongside will be performed. The chosen implementation strategies are:

1. A common strategy consisting of professional audit and feedback.

2. An innovative strategy including a BOS tailored to the barriers and facilitators that are found in a pilot study and professional audit and feedback.

### Preparation

The first part of the study consists of the development of a set of quality indicators extracted from the NVOG guidelines on hypertension in pregnancy. The indicator development will be performed according to the RAND-modified Delphi method [21]. First, key recommendations from the guidelines will be extracted by two or three experts (project leaders). Subsequently, the relevance of all key recommendations for patient's health benefit (clinical relevance) and efficacy will be tested in two rounds among an independent panel of 12 to 15 experts. The opinion leaders of clinical obstetrics, in particular regarding hypertension in pregnancy, as well as members of the NVOG committees on maternal death, guidelines, implementation and quality, quality of care experts, and patients' organisation Stichting HELLP are involved in this expert panel. The key recommendations with the highest scores will be selected and operationalised in measurable elements (process indicators). Based on this set of quality indicators, the BOS for the guidelines on hypertension in pregnancy will be further developed. Moreover, measurement instruments will be developed and the participating hospitals will be informed about the study.

Ethical approval was granted june 12^th ^2008, reference number 2008/138.

### Pilot study

The innovative strategy will be tested in one of the participating hospitals to explore its feasibility.

### Implementation study

An effect and process evaluation will be performed. An effect evaluation of the two strategies will be carried out using the primary and secondary outcome measures. This will be done among pregnant women who develop severe hypertension or preeclampsia and professionals involved in the care for these women. The measurements will be performed in 20 participating hospitals before and after implementation of the different strategies by a medical record search, added with questionnaires among professionals and patients. The medical record search will be done using standardised registration forms. Follow-up of the patients will be six months after delivery.

A process evaluation will be performed to study the feasibility of the two strategies. The extent by which clinicians, nurses, and patients used BOS and the other elements of the strategies (audit and feedback) and their experiences with these elements will be measured. The process information regarding to what extent professionals used BOS will be collected by the application itself. The system will register relevant data of participating professionals and their patients. This will allow us to analyse the relation between the use of BOS and the primary and secondary outcome measures. To measure the experiences with BOS and the other elements of the two strategies, personal interviews will be held among 10 to 15 gynaecologists, nurses, and patients. This will provide insights into possible barriers and facilitators for and satisfaction with using BOS. The possible barriers and facilitators will be quantified with questionnaires among all participating professionals and patients.

For analysis, descriptive as well as regression techniques will be used. A cost effectiveness and cost utility analyses will be performed according to the intention-to-treat principle and from a societal perspective. Cost effectiveness ratios will be calculated using bootstrapping techniques (see economic evaluation).

### Participants

Gynaecologists of 20 Dutch hospitals (university-based, teaching and non teaching) will be invited for participation. Pregnant women who develop severe hypertension or preeclampsia (in accordance to guidelines definitions) in these hospitals will be included. It appears from Dutch data that immigrants are at increased risk for preeclampsia [22]. This is the reason why patient characteristics like age, race, and cultural background will be included in a multilevel analysis. Exclusion criteria are refusal to participate or a diagnosis of lethal fetal congenital abnormalities.

This study will be conducted within the framework of the Otterlo group (a NVOG working group that develops obstetrical guidelines). Representatives of eight academic hospitals and two large teaching hospitals participate in this group. Representatives of another 10 related hospitals will be invited to participate. These hospitals are currently collaborating in the Dutch Perinatal Research Consortium http://www.studies-obsgyn.nl/index.asp.

### Interventions

Innovative strategy including a computerised decision support system. We performed a pilot-study to make an inventory of the barriers and facilitators for adherence to the guidelines on hypertension in pregnancy. Barriers can arise at different stages in the healthcare system at the level of the individual professional (*i.e*., lack of knowledge or skills, resistance against working with protocols); of the healthcare team (*i.e*., lack of knowledge or skills, insufficient collaboration); of the patient (*i.e*., unwillingness, difficult guideline adherence for some patient groupes); of the healthcare organisation (*i.e*., lack of registration/information systems, time restriction for adequate care); and of the wider environment (*i.e*., lack of financial incentives, misuse of guideline in medical disciplinary law) [20]. Barriers can also arise at the level of the innovation (guideline) itself (*i.e*., doubting evidence of the guideline, poor layout, unclear decision tree, unclear content). In a pre-study among 14 gynaecologists in 11 different hospitals (university medical centres, teaching and non-teaching hospitals), we determined which barriers at which level were present regarding the implementation of the guidelines on hypertension in pregnancy. The main barriers proved to be at the level of the guideline itself. A poor layout to use the guideline showed the highest score (64%). Doubt about a clear decision tree behind the guideline scored also high (57%). Furthermore, 36% of the gynaecologists mentioned that 'the guideline could easy be misused in medical disciplinary law,' 'the content of the guideline was unclear,' and that 'there was not enough room to consider patients wishes.' Moreover, 50% of the gynaecologists doubted about the accuracy of certain parts of the guideline. The implementation strategy will be based on these barriers and facilitators. We will use a computerised decision support system, which is a commercial web application developed by Giant-Soft in Leeuwarden, the Netherlands. A similar system has been tested and evaluated using another clinical guideline. The system appeared feasible in clinical practice: most users (95%; 34 of 36) were satisfied with its use and 78% (28 of 36) would prefer to maintain and extend the application to other guidelines [23,24]. In our opinion, BOS can be a solution for the main barriers regarding layout of, and decision tree behind, the guideline. In this way, BOS is tailored to the barriers 'possible misuse in the medical disciplinary law' and 'not enough room to consider patients' wishes.' In this strategy, we will combine BOS with professional audit and feedback (see control intervention).

### Control intervention

The control intervention will consist of professional audit and feedback only. We will perform a retrospective medical record search (audit) for patients with preeclampsia in the 20 hospitals, university hospitals, teaching and non-teaching hospitals. These records will be checked for the extent of adherence to the quality indicators developed from the guideline *Hypertensive Disorders In Pregnancy*. Feedback about the results of the audits will be given in a face-to-face meeting.

## Outcome measures

### Primary outcome

The primary outcome measure for the evaluation of the effectiveness of both strategies is a combined rate of major maternal complications (maternal death, organ specific complications of hypertension, HELLP syndrome, placental abruption).

### Secondary outcomes

Secondary outcome measures for effectiveness are guidelines' adherence rates, fetal death rates, Caesarean delivery rates, and rates of neonatal mortality and morbidity. To measure guidelines' adherence rates, process indicators will be established after selection of key recommendations with the highest scores in the RAND-modified Delphi method.

In the process evaluation, experiences with the implementation strategies are measured. This includes both parameters regarding the participation and presence of the professionals at the feedback meeting and patients in the implementation activities; in particular, the use of the BOS application at patient level as parameters regarding their satisfaction with BOS and the other implementation activities and their meaning about the feasibility (barriers and facilitators) of these activities.

### Sample size

**Figure 1:**
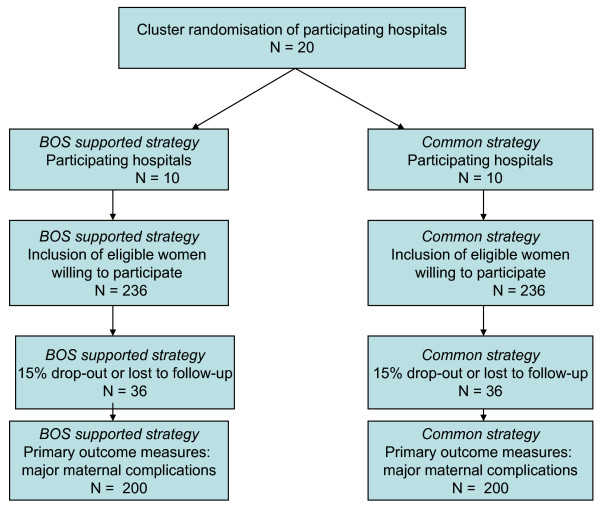
**flowchart randomisation and inclusion**.

The rate of major maternal complications in 216 Dutch patients with severe hypertension or preeclampsia was reported as 34% [7,8]. In 96% of cases of maternal death, care was suboptimal with insufficient treatment of hypertension in about half of these [14]. It is expected that an increased adherence to the guidelines' recommendations will reduce the number of major maternal complications by one-half, *i.e*., from 34% to 17%. Considering an intracluster correlation of 0.05, 20 hospitals will have to be included with approximately 20 patients each in order to get a reliable estimate (alpha = 0.05; power = 0.80) of a 50%-difference (34 verus 17%) in major maternal complications between both groups. If it is assumed that 15% of participants will drop out or be lost to follow up, 472 eligible women will have to be included. It also is assumed that the total number of deliveries in the participating hospitals is 30,000 (during 18 months of inclusion in 20 hospitals) and the incidence of severe hypertension and preeclampsia is 2%. It is estimated that the source

population consists of 600 eligible women. Thus, the expected number of inclusions per month will be 33 in 20 hospitals, *i.e*., one or two per hospital. The minimum required number of inclusions for randomisation per month is 26 [see figure 1 for flowchart].

### Data analysis

To analyse the effectiveness of the two implementation strategies with regard to the primary and secondary outcomes and process indicators, descriptive as well as regression techniques will be used. The analysis will include an elementary head-to-head comparison of the two intervention groups, as well as a multi-level analysis. The latter will incorporate the levels of gynaecologist, patient, and time measurements. The process information regarding to what extent professionals used BOS will allow us to analyse the relation between the use of BOS and the primary and secondary outcome measures. Process information will also give us insight into the experiences of the participants (both satisfaction and feasibility) with the BOS and the other implementation activities.

### Economic evaluation

The economic evaluation (cost effectiveness and cost utility analysis) will be performed according to the intention-to-treat principle and from a societal perspective. Healthcare costs (costs of hospitalisation, medication), costs in other sectors, patient and family costs, and costs of production losses are included. Quality adjusted life years (QALYs) are used as the main health outcome measure for the cost-utility analysis. Incremental cost effectiveness ratios (ICER) will be estimated using bootstrapping techniques and graphically presented on cost effectiveness planes. Sensitivity analysis on the most important assumptions will be performed in order to assess the robustness of the results.

QALYs will be calculated as the area under the health utility profile, with straight-line interpolation between utilities at each follow up. Utilities will be obtained using the EuroQol (EQ-5D) instrument. Health states will be estimated using reference values from a representative Dutch sample. ICER will be estimated using bootstrapping techniques and graphically presented on cost effectiveness planes. Sensitivity analysis on the most important assumptions will be performed in order to assess the robustness of the results.

The input of the implementation strategies will be assessed by collecting volumes of consumed resources and multiplying these by the price of each resource unit (market or guideline price). The output of the implementation strategies will be determined by the guidelines adoption rate, measured at fixed intervals during the study and the speed of adoption. Welfare losses will be determined by empirically established diffusion curves (one diffusion curve per strategy) and quantified by calculating the welfare change (monetarized variation in indicators/benchmarks compared to guideline) at the different time intervals adjusted for the complement of the adoption rate at a particular time interval. The decision criterion on which the efficient implementation strategy will be selected is the minimum welfare loss compared to the guideline programme over time (dynamic efficiency). The impact of variable(s) uncertainty on the decision criterion will be evaluated by sensitivity analyses.

## Discussion

This study addresses an important problem, because hypertensive disease in pregnancy is the main cause of maternal mortality in the Netherlands and analysis of these deaths suggests 96% received substandard care. This indicates that there is room for improvement of the management of care for this group of pregnant women [14].

Clinical guidelines aim to promote evidence-based practice, improve patient outcome, and allow more efficient use of resources [25]. In a previous study of the NVOG guideline on intra-uterine insemination, yet to be publicised, it was demonstrated that there was a favourable cost effectiveness ratio regarding adherence to the main indicators of that guideline. Similar results are assumed for the NVOG guidelines on hypertension in pregnancy and chronic hypertension.

This study will also allow us to ascertain which elements of a multifaceted strategy can be particularly associated with successful implementation of the guidelines on hypertension in pregnancy in the hospital setting. The results of our study can contribute to more knowledge about the effectiveness of a health information technology intervention on guideline implementation.

## Competing interests

The authors declare that they have no competing interests.

## Authors' contributions

SL drafted the manuscript. MW conceived of the study, and participated in its design and coordination, and helped to draft the manuscript. AF conceived of the study, and participated in its design and coordination. HS conceived of the study, and participated in its design and coordination. HW conceived of the study, and participated in its design and coordination. ES conceived of the study, and participated in its design and coordination. MH conceived of the study, and participated in its design and coordination. RH conceived of the study, participated in its design and coordination, and helped to draft the manuscript. VH participated in the design of the study and performed the statistical analysis. MT conceived of the study, and participated in its design and coordination and helped to draft the manuscript. All authors read and approved the final manuscript.

## Ethical approval

Ethical approval was granted june 12^th ^2008.
